# Effects of Coagent Functionalities on Properties of Ultrafine Fully Vulcanized Powdered Natural Rubber Prepared as Toughening Filler in Rigid PVC

**DOI:** 10.3390/polym13020289

**Published:** 2021-01-18

**Authors:** Yiting Lin, Lunjakorn Amornkitbamrung, Phattarin Mora, Chanchira Jubsilp, Kasinee Hemvichian, Apinan Soottitantawat, Sanong Ekgasit, Sarawut Rimdusit

**Affiliations:** 1 Research Unit in Polymeric Materials for Medical Practice Devices, Department of Chemical Engineering, Faculty of Engineering, Chulalongkorn University, Bangkok 10330, Thailand; yitinglin929@gmail.com (Y.L.); a.lunjakorn@gmail.com (L.A.); mootoo_punch@hotmail.com (P.M.); 2 Department of Chemical Engineering, Faculty of Engineering, Srinakharinwirot University, Nakhonnayok 26120, Thailand; chanchira@g.swu.ac.th; 3 Thailand Institute of Nuclear Technology, Nakhonnayok 26120, Thailand; kasineeh@yahoo.com; 4 Department of Chemical Engineering and Center of Excellence in Particle Technology, Faculty of Engineering, Chulalongkorn University, Bangkok 10330, Thailand; apinan.s@chula.ac.th; 5 Sensor Research Unit, Department of Chemistry, Faculty of Science, Chulalongkorn University, Bangkok 10330, Thailand; sanong.e@chula.ac.th; 6 Research Network NANOTEC-CU on Advanced Structural and Functional Nanomaterials, Chulalongkorn University, Bangkok 10330, Thailand

**Keywords:** ultrafine fully vulcanized powdered natural rubber, radiation vulcanization, coagent, toughening filler, PVC

## Abstract

Ultrafine fully vulcanized powdered natural rubber (UFPNR) has a promising application as a renewable toughening modifier in polymer matrices. In this work, the effects of acrylate coagents, which had different amounts of functional groups, on properties of UFPNR produced by radiation vulcanization and spray-drying was systematically investigated for the first time. Dipropylene glycol diacrylate (DPGDA), trimethylol propane trimethaacrylate (TMPTMA), and ditrimethylol propane tetraacrylate (DTMPTA) were used as coagents with two, three, and four acrylate groups, respectively. The radiation in the range of 250 to 400 kGy and coagent contents of up to 11 phr were used in the production process. Physical, chemical, and thermal properties of the UFPNR were characterized by swelling analysis, scanning electron microscopy, infrared spectroscopy, thermogravimetric analysis, and differential scanning calorimetry. The properties of UFPNR produced by using different type and content of coagents were compared and discussed. The results revealed that UFPNR with the smallest particle size of 3.6 ± 1.1 μm and the highest thermal stability (Td5 = 349 °C) could be obtained by using DTMPTA, which had the highest amount of functional group. It was proposed that the coagent with the greater number of acrylate groups enhanced the crosslinking of natural rubber as it had more reactive groups. Finally, an application of UFPNR as a toughening filler in rigid PVC was demonstrated with 34% improvement of impact strength.

## 1. Introduction

Natural rubber (NR) extracted from the *Hevea brasiliensis* tree is a natural and renewable material and a main component of cis-1,4-polyisoprene polymer. NR in its original form has a sticky characteristic and non-elastic properties [1,2,3,4]. To improve NR properties for extended applications, vulcanization has been applied to crosslink NR molecules, which makes NR heat stable and elastic [1]. Vulcanization of NR can be done by three main techniques using sulfur, peroxide, or radiation. Incorporation of functionalized nanomaterials is another approach to NR vulcanization [5]. Radiation vulcanization is becoming more favorable than the two conventional techniques as it possesses many promising advantages, namely a high degree of crosslinking, no oxidative degeneration in polymer, less energy consumption as it is operated at room temperature, and inherently clean technology [6,7]. In the process of radiation vulcanization, NR molecules absorb radiation energy, in which NR radicals are generated by radiolysis of NR molecules. Then, the radicals recombine with each other, forming a crosslinking structure in NR particles [1]. After irradiation, the vulcanized NR can be produced into powder form by a spray-drying process [8]. The obtained product is called ultrafine fully vulcanized powdered natural rubber (UFPNR). The material has demonstrated a promising application as a toughening modifier in polymer matrix [7]. Research and development on UFPNR is being continually carried out to improve its properties for use as a toughening modifier in polymer matrices at the industrial level, with competitive performance compared to petroleum-based ultrafine fully vulcanized powdered rubber (UFPR) from styrene-butadiene, nitrile–butadiene, etc. [9,10,11,12,13,14,15,16,17,18,19].

In radiation vulcanization, the crosslinking of NR can be further enhanced by the addition of monofunctional and polyfunctional monomers, also called coagents [6]. The use of coagents can help to obtain vulcanized NR with desired properties at lower doses of radiation, which consequently reduces the degradation of NR induced by high radiation dose [20]. Coagents can boost the vulcanization efficiency as they are highly reactive toward free radicals. Organic molecules with acrylate groups, i.e., trimethylolpropane trimethaacrylate, ethyleneglycol dimethacrylate, and phenoxy ethyl acrylate are one of the most widely used coagent in radiation vulcanization of NR and they are proven to enhance crosslink density of NR during radiation vulcanization [6,21]. In addition, acrylate coagents, i.e., ethyleneglycol dimethacrylate and pentaerythritol triacrylate, have also been used for radiation vulcanization of synthetic rubbers. It was found that they were used for improving physico-mechanical properties during radiation vulcanization of acrylic rubber/styrene butadiene rubber blend at low radiation dose [20]. Although a number of acrylate coagents were found to enhance crosslinking of NR during radiation vulcanization, a detailed and systematic study comparing the coagents has not been carried out. In particular, the effect of the number of acrylate groups on properties of UFPNR produced by radiation vulcanization and spray-drying has not yet been studied.

In this work, we systematically investigated the effects of acrylate coagents, which had different numbers of functional groups, on the properties of UFPNR produced by radiation vulcanization and spray-drying. Dipropylene glycol diacrylate, trimethylol propane trimethaacrylate, and ditrimethylol propane tetraacrylate were used as coagents with two, three, and four acrylate groups, respectively. Physical, chemical, and thermal properties of UFPNR were characterized by swelling analysis, scanning electron microscopy, infrared spectroscopy, thermogravimetric analysis, and differential scanning calorimetry. The effect of radiation dose on the properties of UFPNR was studied in the range of 250 to 400 kGy. The properties of UFPNR produced by using different types and contents of coagents were compared and discussed. Finally, application of UFPNR as a toughening filler in PVC was demonstrated in terms of impact strength improvement.

## 2. Materials and Methods

### 2.1. Materials

**Figure 1:**
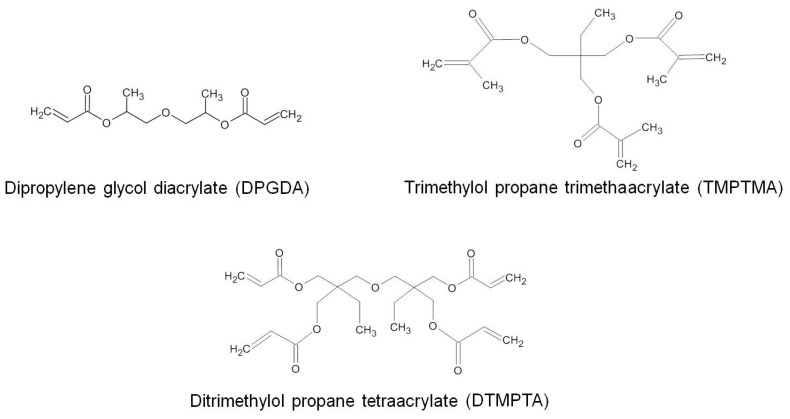
Molecular structures of DPGDA, TMPTMA, and DTMPTA coagents.

NR latex containing 60 wt.% of solid content was supplied by the Rubber Research Institute of Thailand. Acrylate coagents, i.e., dipropylene glycol diacrylate (DPGDA, purity > 75%), trimethylol propane trimethaacrylate (TMPTMA, purity > 75%), and ditrimethylol propane tetraacrylate (DTMPTA, purity > 75%) were purchased from Tokyo Chemical Industry Co., Ltd. (Tokyo, Japan). The molecular structures of acrylate coagents are presented in Figure 1. Polyvinyl chloride (PVC, K 66) was provided by Thai Plastic and Chemicals Co., Ltd. (Bangkok, Thailand). All chemicals were used as received without further purification.

### 2.2. Preparation of UFPNR

NR latex containing 60 wt.% of solid content was diluted to 20 wt.% of solid content with distilled water, followed by the addition of each acrylate coagent with different amounts in the range of 1 to 11 phr. The mixtures were stirred for 15 min, followed by radiation vulcanization using an electron beam accelerator module MB 10–50, facilitated by the Thailand Institute of Nuclear Technology, with 10 meV and 50 kW of output power. The radiation dose was varied in the range of 250 to 400 kGy. Then, the radiated samples were dried by using a Buchi Mini Spray Dryer Model B-290 (Flawil, Switzerland) with an inlet temperature of 150 °C. The flow meter valve was set to 667 L/h. The pump output was set to 4.5 mL/min of feed flow pumping into the nozzle. The UFPNR was obtained at the bottom part of the cyclone unit.

### 2.3. Preparation of UFPNR-Filled PVC

PVC was dry-mixed with various contents (0, 5, 8, 10, and 15 phr) of UFPNR. The mixtures were blended in a two-roll miller at 180 °C for 4 min, yielding PVC/UFPNR sheets. The sheets were then compressed in a compression molder at 180 °C for 4 min and cut into the desired shape following the ASTM D256 standard. The dimensions of each specimen was 64 × 12.7 × 3.2 mm. The depth under the notch of each specimen was 2.5 ± 0.05 mm with an angle of 44.5 ± 0.5°.

### 2.4. Characterization

Swelling behavior of UFPNR was analyzed by dissolution of approximately 0.100 g of the sample in 20 mL toluene at room temperature for 24 h. The dried weight of UFPNR sample (W_1_) was recorded, and the sample was then immersed in toluene (ρ_s_ = 0.87 g/cm^3^, V_1_ = 106.5 cm^3^/mol) for 24 h. After that, the weight of swollen UFPNR sample (W_2_) was immediately recorded, followed by drying of the sample in a vacuum oven at 70 °C for 24 h. Finally, the weight of sample (W_3_) was recorded after drying. The swelling ratio (Q), gel fraction, molecular weight between the crosslink (M_c_), and crosslinking density (CLD) were calculated according to Equations (1)–(4), respectively [22,23]. The density of rubber (ρ_r_) is 0.91 g/cm^3^. φ_r_ is the volume fraction of the polymer in the swollen stage and χ_12_ is the polymer–solvent interaction parameter, which was equal to 0.393. N is the Avogadro number of 6.02214179 × 10^23^.

(1) $$Q=\;\frac{\left(W_{2}-W_{1}\right)/\rho_{s}}{W_{1}/\rho_{r}}$$

(2) $$\mathrm{Gel}\;\mathrm{fraction}=\frac{W_{3}}{W_{1}}$$

(3) $$M_{c}=\frac{-\rho_{r}\;V_{1}\left({\varphi_{r}}^{1/3}-\frac{\varphi_{r}}{2}\right)}{\mathrm{Ln}\left(1-\varphi_{r}\right)+\varphi_{r}+\chi_{12}\varphi_{r}^{2}}:\mathrm{where}\;\varphi_{r}=\;\frac{1}{1+Q}.$$

(4) $$\mathrm{CLD}=\frac{\rho_{r}N}{M_{c}}$$

Scanning electron micrographs of UFPNR were observed by using a JEOL JSM-6400 scanning electron microscope (SEM) (Tokyo, Japan)with an accelerating voltage of 3 kV. The UFPNR samples were coated with thin gold by using a JEOL ion sputtering device (model JFC-1200). The particle size was measured by using the Image J program.

The molecular structure of UFPNR was examined by using a Spectrum GX FT-IR spectrometer (Perkin Elmer, Waltham, MA, USA) with an attenuated total reflection (ATR) accessory. The analysis was carried out at a scan range of 4000–400 cm^−1^ with a total scan of 32 and resolution of 4 cm^−1^.

The thermal stability of UFPNR was analyzed using a thermogravimetric analyzer (TGA, model TGA1 Module, Mettler-Toledo, Columbus, OH, USA) with the weight of each sample in the range of 8 to 12 mg. The measurements were carried out by heating from 25 °C to 850 °C with a heating rate of 20 °C/min under a nitrogen atmosphere and with a flow rate of 50 mL/min.

A differential scanning calorimeter (DSC, model DSC1 Module, Mettler-Toledo) was used to determine the glass transition temperature of UFPNR. The sample mass of 5 to 10 mg was heated from −100 °C to 25 °C at a heating rate of 10 °C/min under a nitrogen atmosphere and cooling system by using a liquid nitrogen.

Izod impact strength testing of PVC/UFPNR was carried out following the standard method of ASTM D256. The specimen was clamped into the pendulum impact test fixture with the notch side facing the striking edge of the pendulum on the Izod impact tester.

## 3. Results

### 3.1. Effect of Radiation Dose on Swelling Behavior of UFPNR

**Figure 2:**
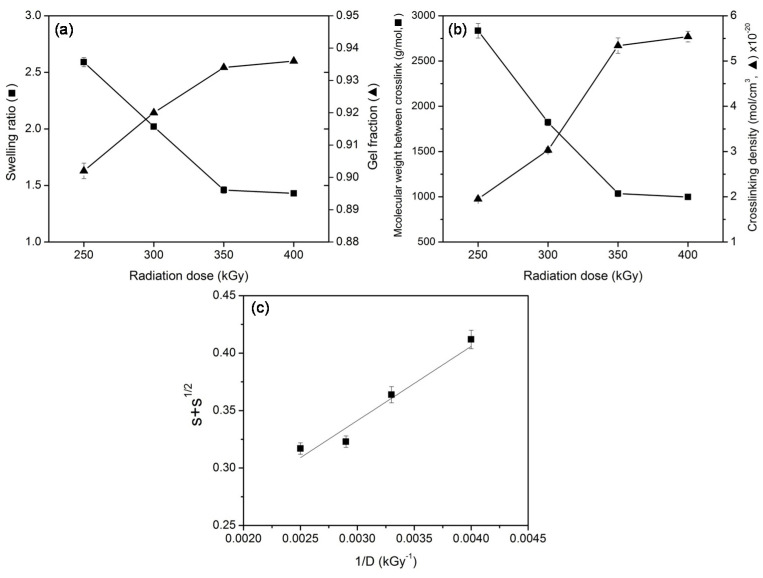
Swelling ratio and gel fraction of UFPNR (**a**), molecular weight between the crosslink and crosslinking density of UFPNR (**b**), and Charlesby–Pinner plot of UFPNR prepared with different radiation doses without using coagent (**c**).

The effect of radiation dose on swelling behavior of UFPNR was studied. UFPNR was prepared with different radiation doses from 250 to 400 kGy without using coagent. The swelling ratio, gel fraction, molecular weight between the crosslink, and crosslink density were determined, and the results are presented in Figure 2. It can be observed in Figure 2a that the increased radiation dose resulted in the decreased swelling ratio of UFPNR, while the gel fraction of UFPNR became higher with increases in the radiation dose. The enhancement of the gel fraction and the diminishing swelling ratio at higher radiation dose were due to the increase in insoluble polymer fraction in UFPNR [24]. This phenomenon was attributed to the increased inter-molecular crosslink in UFPNR [7,25]. The plot in Figure 2b confirmed the increased crosslinking of UFPNR at higher radiation dose, as it showed a decrease in molecular weight between the crosslink of UFPNR. Based on the Flory–Rehner theory, the molecular weight between the crosslink decreased as the length of polymer chain became shorter when it was more crosslinked [22,23]. At higher radiation dose, more free radicals were generated in NR as it absorbed more energy [26]. Consequently, the crosslinking reaction was promoted, confirming by the increased crosslinking density of UFPNR, as presented in Figure 2b. In overall, these results implied that the higher dose of radiation enhanced the crosslinking in UFPNR.

During the irradiation process, free radicals formed on the electron beam of the NR, caused crosslinking and scission of the polymer chain. The net effect of radiation on NR depended on the ratio between the degree of chain crosslinking and chain scission, which could be determined by using the Charlesby–Pinner equation as follows [27]:

(5) $$s+s^{1/2}\;=\frac{p}{q}+\;\frac{1}{qu_{1}D}$$

where *s* is the soluble fraction (*s* = 1 − gel fraction), *p* is the chain scission probability, *q* is the chain crosslinking probability, *u*_1_ is the number-average degree of polymerization, and *D* is the radiation dose [28]. The plot between 1/*D* and *s* + *s*^1/2^ is presented in Figure 2c. The value of *p*/*q* = 0.147 could be obtained from the y-interception from linear fitting, and it implied that NR predominantly underwent crosslinking rather than scission in the range of radiation doses from 250 to 400 kGy [28].

### 3.2. Effect of Radiation Dose on Properties of UFPNR

**Figure 3:**
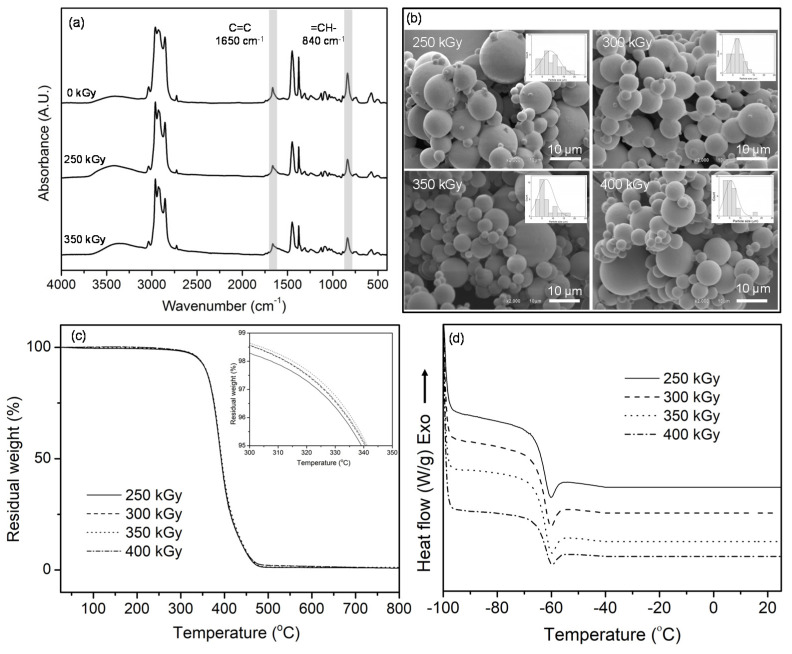
Infrared spectra (**a**), SEM micrographs (**b**), TGA curves (**c**), and DSC curves (**d**) of UFPNR prepared with different radiation doses without using coagent.

In order to confirm the crosslinking of UFPNR upon the radiation, the molecular properties of UFPNR were analyzed by using infrared spectroscopy. As shown in Figure 3a, infrared spectra of un-radiated and radiated UFPNR had a similar characteristic, which agreed well with previous reports [6,7]. The effect of radiation dose was observed as the intensities of the peaks at 1650 and 840 cm^−1^, corresponding to C=C stretching vibration and =CH—out of plane bending of NR, respectively, decreased with increased radiation dose. The result suggested that the double bond was consumed as NR was crosslinked and transformed into a networking structure upon the radiation process [6,7,29]. The crosslinking mechanism of NR upon radiation has been proposed in a previous report [6]. The effect of radiation dose on morphology of the UFPNR was observed by using SEM. As can be seen in Figure 3b, the average particle size of UFPNR gradually decreased from 8.8 ± 4.2 to 7.1 ± 2.5, 5.7 ± 2.9, and 6.8 ± 3.4 μm, with increased radiation dose from 250 to 400 kGy. Moreover, the UFPNR particles became less aggregated at higher radiation dose. Decreased particle size and less aggregation were due to the fact that the polymer chains of NR were more crosslinked and packed into a stable particle form at higher radiation dose. It should be noted that the large particle size distribution of UFPNR might be due to the large particle size distribution of NR particles in the latex, which was used as a starting material for UFPNR production [2,4]. Separation of large NR particles from small NR particles in the latex could be one of the good ways to improve this situation.

The effect of radiation dose on the thermal properties of UFPNR was analyzed by using TGA and DSC. The degradation temperature at 5% weight loss (Td5) of the UFPNR, as depicted in Figure 3c, was not significantly changed with increased radiation dose. The Td5 value was in the narrow range of 339 to 341 °C with increased dose from 250 to 400 kGy. In the case of the glass transition temperature of UFPNR, it was not significantly altered with increased radiation dose. This result agreed well with the result in Figure 2b, which showed a narrow range of crosslinking density formed as result of irradiation of NR [24]. As presented in Figure 3d, the glass transition temperature of UFPNR was around −60 to −61 °C, which was in the same range as in a previous report [30]. According to the results, the radiation dose at 350 kGy is appropriate to vulcanize NR and to obtain UFPNR, which has a smaller particle size with less aggregation.

### 3.3. Effect of Coagent on Swelling Behavior of UFPNR

**Figure 4:**
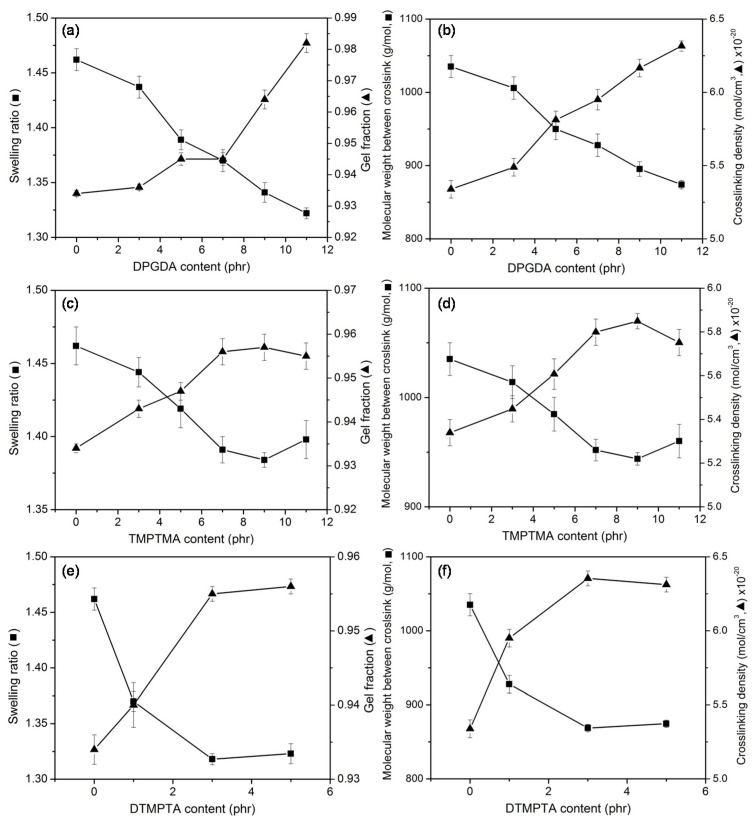
Swelling ratio and gel fraction (**a**,**c**,**e**), and molecular weight between the crosslink and crosslinking density (**b**,**d**,**f**) of UFPNR prepared with different types and contents of coagent.

The reactivity of coagent plays an important role during the irradiation process. Coagent can either suppress non-network-forming side reactions or generate additional crosslinks to increase the crosslinking density of the polymer network [6], and physical and thermal properties of vulcanized polymer are strongly affected by coagent [25]. In this work, the effect of acrylate coagents, which had different numbers of functional groups, on properties of UFPNR was investigated. DPGDA, TMPTMA, and DTMPTA were used as coagents with di-, tri-, and tetra-acrylate groups, respectively. The content of coagents was in the range of 1–11 phr. The radiation dose was fixed at 350 kGy. The swelling ratio and gel fraction of UFPNR with regard to the type and content of coagents are shown in Figure 4. For all types of coagents, the results demonstrated that when the content of coagents increased, there was a decrease in swelling ratio and increase of gel fraction of the UFPNR. A decrease in molecular weight between the crosslink and increase of crosslinking density with an increase in coagent content could also be observed. These results confirmed that crosslinking of the UFPNR was further enhanced by using coagents, and the degree of enhancement was similar for all types of coagents. However, the excess contents, i.e., 9 and 11 phr of DPGDA, resulted in the precipitation of latex after irradiation. In the case of TMPTMA and DTMPTA, their excess contents of 11 and 5 phr, respectively, caused the phase separation before irradiation process, as TMPTMA and DTMPTA were not soluble in the NR solution. The phase separation occurred due to the domain of the high local concentration of these coagents. Consequently, the reactivity of the coagents decreased according to the steric hindrance effect [31]. A slight decrease of crosslinking density could be observed when high contents of coagents were used, especially for TMPTMA and DTMPTA. To avoid the effect of precipitation, DPGDA, TMPTMA, and DTMPTA with contents of 3 phr were selected for use in the next experiment.

### 3.4. Effect of Coagent on UFPNR Properties

**Figure 5:**
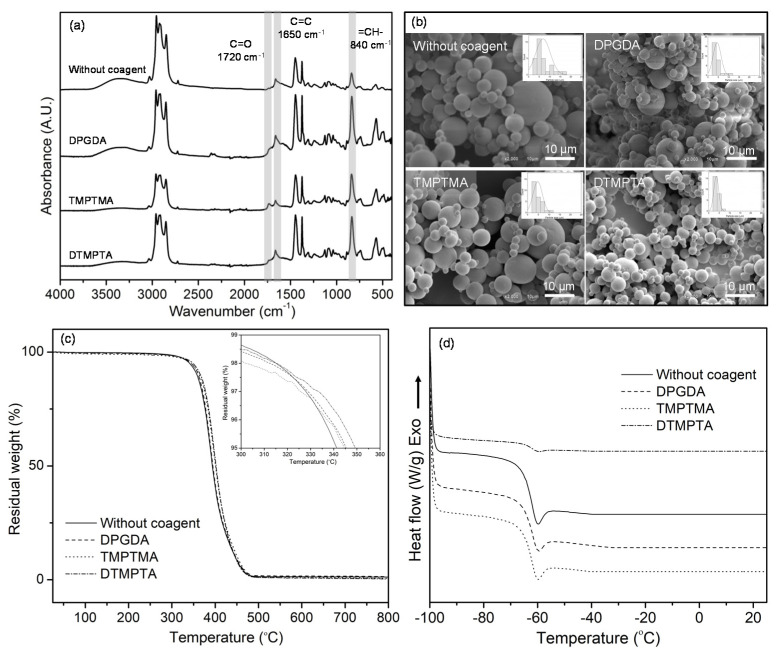
Infrared spectra (**a**), SEM micrographs (**b**), TGA curves (**c**), and DSC curves (**d**) of UFPNR prepared with each coagent at 3 phr.

**Figure 6:**
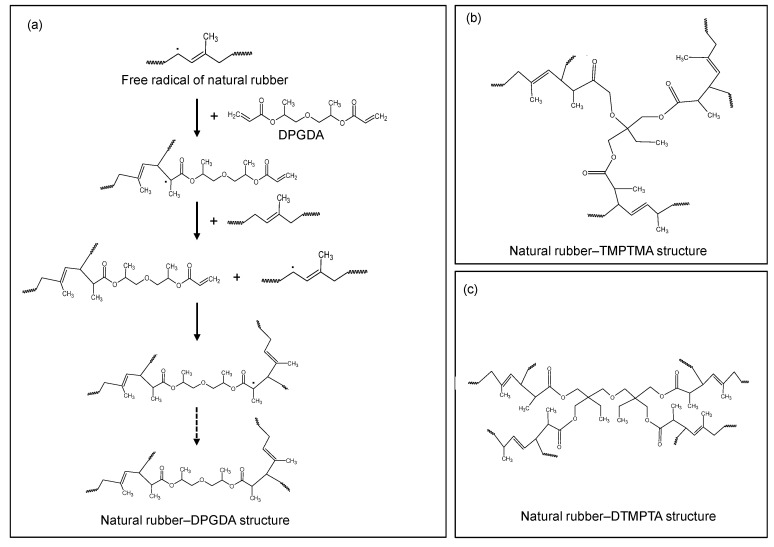
Formation mechanism (**a**) and network structures (**a**–**c**) of natural rubber formed by using coagents with different functionalities.

The molecular properties of UFPNR with regard to coagent functionality were investigated by using infrared spectroscopy. Infrared spectra in Figure 5a revealed that coagents altered the molecular structure of UFPNR. For the UFPNR produced with coagent, the intensity of peaks at 1650 and 840 cm^−1^, corresponding to C=C stretching vibration and =CH– out of plane bending of NR, respectively, tended to become more than those without coagent, which suggested that the double bond of NR might not be much consumed upon irradiation. This might be due to the fact that the double bond of the coagent might be consumed instead. As an example, the reaction mechanism for the network formation of NR and DPGDA coagent was proposed, as seen in Figure 6a. Radicals in the NR chains were formed by an electron beam, and with the presence of coagents, the free radicals could react with the double bonds of the coagents, which were highly reactive toward free radicals [6]. As shown in Figure 6a–c, three-dimensional network structures of NR were formed with coagents, which acted as multi-modal crosslinking centers, binding NR chains together. As presented in Figure 5a, the peak at 1720 cm^−1^, corresponding to C=O stretching vibrations of coagent molecules, was observed in the UFPNR structure.

The effects of coagent functionality on the morphology of UFPNR were observed by using SEM. As can be seen in Figure 5b, the average particle size of the UFPNR produced without coagent systematically decreased from 5.7 ± 2.9 to 4.4 ± 2.0, 4.7 ± 2.1, and 3.6 ± 1.1 μm, with the use of DPGDA, TMPTMA, and DTMPTA, respectively. The results suggested that coagent with higher amounts of acrylate functional group enhanced the crosslinking of NR, as it had more reactive groups. Thus, polymer chains of NR were more crosslinked and packed into a smaller particle form of the UFPNR.

The effect of coagent functionality on the thermal properties of UFPNR was analyzed by using TGA and DSC. As presented in Figure 5c, the degradation temperature at 5% weight loss of UFPNR produced without coagent systematically increased from 341 to 343, 345, and 349 °C, with the use of DPGDA, TMPTMA, and DTMPTA, respectively. The enhancement of thermal stability was due to the higher molecular crosslinking of NR. These results supported the findings from SEM results, confirming that the coagent with greater numbers of acrylate functional groups enhanced the crosslinking of NR. However, the glass transition temperature (Tg) of UFPNR was not significantly altered with coagent functionality, i.e., Tg around −60 to −61 °C, as can be observed in Figure 5d.

### 3.5. Application of UFPNR as Toughening Filler in PVC

**Figure 7:**
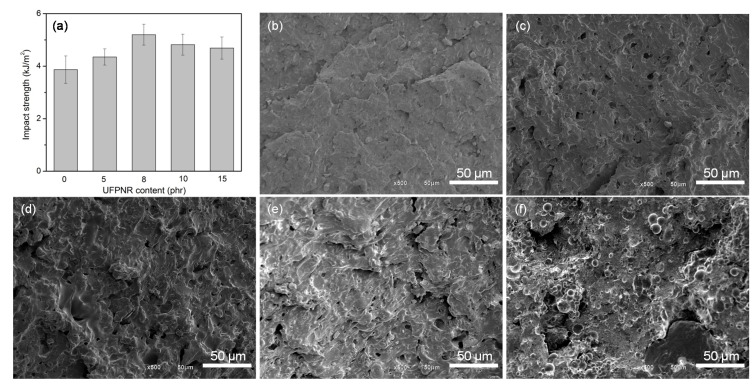
Impact strength of PVC prepared with different contents of UFPNR (**a**), and SEM micrographs of surface fracture of PVC prepared with 0 (**b**), 5 (**c**), 8 (**d**), 10 (**e**), and 15 (**f**) phr UFPNR.

An application of UFPNR as a toughening filler in PVC was demonstrated. Rigid PVC has a limitation of low notch impact strength. It was speculated that UFPNR could improve the impact resistance of rigid PVC. The UFPNR prepared by using 3 phr DTMPTA with radiation dose of 350 kGy was used for the application. The results in Figure 7a revealed that the impact strength of the PVC could be improved for 12.52 and 34.24% with the UFPNR addition of 5 and 8 phr, respectively. The maximum impact strength of UFPNR-toughened PVC was 5.2 kJ/m^2^, which was comparable to the impact strength of PVC toughened by using UFPR made from synthetic rubber [32]. The increased impact strength of PVC corresponded to the toughening effect of the UFPR in the PVC matrix. It is well known that the energy adsorption mechanism of a rubber particle consists of crazing and deformation of the matrix, which is involved in the toughening mechanism [9,32]. The SEM micrographs of the fracture surface of neat PVC and PVC loaded with different contents of the UFPNR are presented in Figure 7b–f. The results showed that PVC loaded with UFPNR had a rougher surface fracture than that of neat PVC. This implied that neat PVC had a brittle fracture mechanism [33]. With the addition of UFPNR content of up to 8 phr, a ductile fracture mechanism could be observed in the fracture surface of PVC, which was spanned by some drawn elongated fibrils. The elongated fibrils maintained the structural integrity of the fractured part by bridging the two opposite surface crazes transmuted into the ductile fracture. Ductile fracture dissipated a high value of impact strength and was associated with extensive shear yielding, which spread over a large volume of part of the fracture surface. However, the UFPNR with excess contents of 10 and 15 phr caused a reduction of impact strength improvement to 24.62 and 21.31%, respectively. The overloaded content of the toughening modifier resulted in a crack due to the decreased dispersion of the UFPNR in the PVC matrix [34,35].

## 4. Conclusions

UFPNR was successfully prepared by radiation vulcanization and spray drying. With increased radiation dose from 250 to 400 kGy, the crosslinking density of UFPNR was enhanced, and the particle size was reduced from 8.8 ± 4.2 to 6.8 ± 3.4 μm. The crosslinking of UFPNR was further enhanced by using coagents. UFPNR, with the smallest particle size of 3.6 ± 1.1 μm and highest thermal stability (Td5 = 349 °C), could be obtained by using DTMPTA, which had the greatest number of acrylate groups. The results suggested that coagents with greatest numbers of acrylate functional groups enhanced the crosslinking of NR as it had more reactive groups. Lastly, application of UFPNR as a toughening filler in rigid PVC was demonstrated. The highest impact strength improvement of 34% was achieved by incorporation of 5 phr UFPNR in PVC.

## Data Availability

The data presented in this study are available on request from the corresponding author.
